# Genetic analysis of three maize husk traits by QTL mapping in a maize-teosinte population

**DOI:** 10.1186/s12864-021-07723-x

**Published:** 2021-05-26

**Authors:** Xiaolei Zhang, Ming Lu, Aiai Xia, Tao Xu, Zhenhai Cui, Ruiying Zhang, Wenguo Liu, Yan He

**Affiliations:** 1 Quality and Safety Institute of Agricultural Products, Heilongjiang Academy of Agricultural Sciences, Harbin, 150086 China; 2 Maize Research Institute, Jilin Academy of Agricultural Sciences, Gongzhuling, 136100 China; 3 Sanya institute of China Agricultural University, Sanya, 572025 China; 4 Tieling Academy of Agricultural Sciences, Tieling, 112000 China; 5 College of Biological Science and Technology, Liaoning Province Research Center of Plant Genetic Engineering Technology, Shenyang Key Laboratory of Maize Genomic Selection Breeding, Shenyang Agricultural University, Shenyang, 110866 China

**Keywords:** Maize, Teosinte, Husk, QTL

## Abstract

**Background:**

The maize husk consists of numerous leafy layers and plays vital roles in protecting the ear from pathogen infection and dehydration. Teosinte, the wild ancestor of maize, has about three layers of small husk outer covering the ear. Although several quantitative trait loci (QTL) underlying husk morphology variation have been reported, the genetic basis of husk traits between teosinte and maize remains unclear.

**Results:**

A linkage population including 191 BC_2_F_8_ inbred lines generated from the maize line Mo17 and the teosinte line X26–4 was used to identify QTL associated with three husk traits: i.e., husk length (HL), husk width (HW) and the number of husk layers (HN). The best linear unbiased predictor (BLUP) depicted wide phenotypic variation and high heritability of all three traits. The HL exhibited greater correlation with HW than HN. A total of 4 QTLs were identified including 1, 1, 2, which are associated with HL, HW and HN, respectively. The proportion of phenotypic variation explained by these QTLs was 9.6, 8.9 and 8.1% for HL, HN and HW, respectively.

**Conclusions:**

The QTLs identified in this study will pave a path to explore candidate genes regulating husk growth and development, and benefit the molecular breeding program based on molecular marker-assisted selection to cultivate maize varieties with an ideal husk morphology.

## Background

Maize (*Zea mays ssp. mays*) is one of the most important cereal and forage crops worldwide. The most effective way for ensuring food supply is to improve maize yield [1]. As a leaf-like tissue covering outside of the ear, the husk plays important biological functions in warranting maize yield. Similar to foliar leaves, the husk can produce carbohydrates through photosynthesis process [2]. In addition, the husk nurseries and protects the ear from pathogen infection, birds and pests attack [3–5]. Moreover, the husk maintains appropriate moisture of kernel growth and prevents ear dehydration [6–14]. Therefore, the proper husk-related traits, i.e., HL, HW and NHL, serve as the critical factors influencing the rate of kernel dehydration after physiological maturity [2, 15–18].

Several recent studies have been conducted to understand the genetic basis of husk morphology [9, 13, 14]. The first QTL mapping about husk traits can be traced back to 2003 related to husk tightness [9]. In a F_2:3_ population, QTLs of husk tightness located on chromosome 1S, 1 L, 3 L and 7 L [9]. In 2016, the first genome wide association study (GWAS) for NH and husk weight were performed using 3 K SNP markers and identified a total of 24 and 29 SNPs associated with HN and husk weight, respectively [19]. At the same year, our group also performed GWAS using a larger scale of population with higher marker density (508 lines with 0.5 million of SNP markers) [13]. Under the stringent threshold *P* < 1.04 × 10^− 5^, nine variants significantly associated with HN, HW and HL were identified [13]. In 2018, the linkage mapping integrated with GWAS revealed five candidate genes related to HL and HN [14]. In 2020, utilizing denser marker (1.25 million) coupled with advanced statistical method, the other five candidate genes associated with HN and HT were detected [2]. Overall, these studies have unambiguously addressed that husk traits are complex and genetically controlled by multiple genes.

Teosinte (*Zea mays ssp. parviglumis*) is the wild progenitor of maize [20–22]. It exhibits significant resistance to cold [23, 24], drought [23], waterlogging [25, 26], pests [27] and diseases [27]. Maize-teosinte populations have been emerged as the ideal materials to identify important genes or QTLs related to multifaced maize traits. In addition, it is helpful to reveal the genetic mechanism of maize adapting to domestication and facilitate continued improvement of maize yield and quality [28–32]. In this study, we utilized a maize-teosinte population (MX) to analyze the genetic basis of three phenotypic husk traits, including HL, HN and HW. In addition, we positioned the large-effect QTL intervals using the bin map and predicted candidate genes associated with husk traits. A total of 4 QTLs were mapped out and 6 candidate genes were identified.

## Results

### Phenotypic variation and heritability of husk traits

**Fig. 1:**
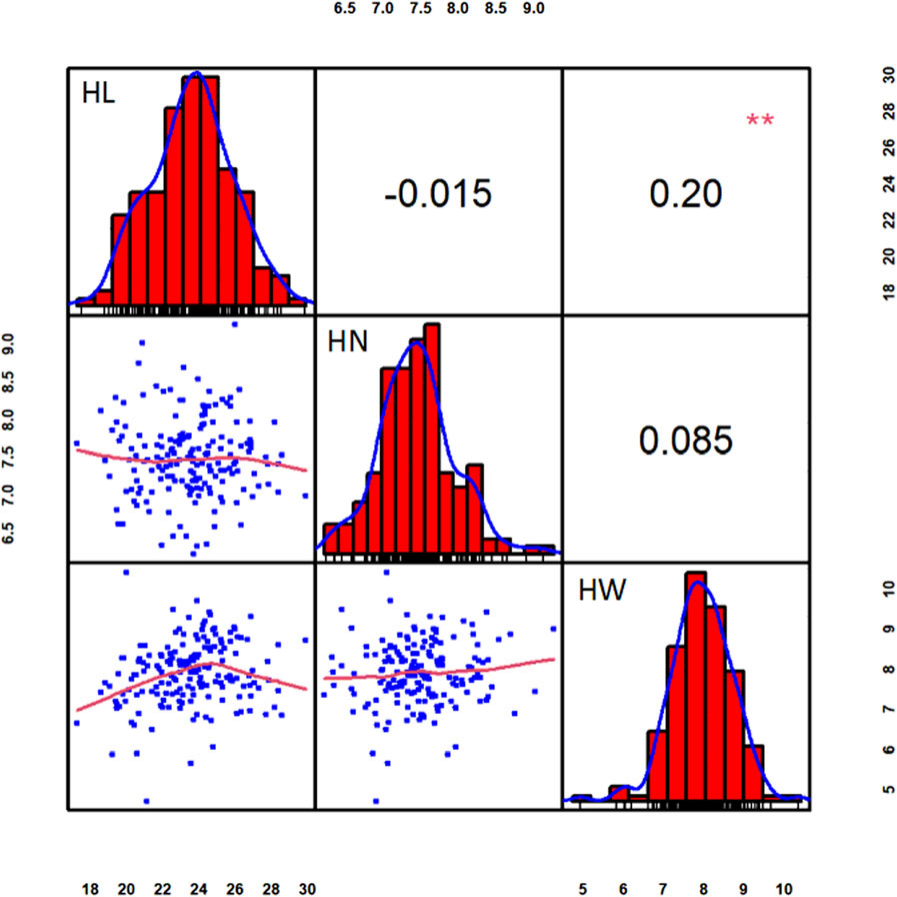
Frequency distributions and correlation coefficients of three husk traits using BLUP values. Plots along diagonal line depict phenotypic distribution of each trait. Values above diagonal line are Pearson’s correlation coefficients between traits. Plots below diagonal line are scatter plots of compared traits. **Significant at *P* ≤ 0.01

The phenotypic variation and heritability of three husk traits in the parental line Mo17 and the recombinant inbred line (RIL) population in three environments were summarized in Table 1. Analyses of the best unbiased linear predictive (BLUP) values revealed that there was a broad range of phenotypic variation while the mean values were close to Mo17-parent value for all the three traits (Table 1). The three husk traits exhibited continuous and approximately normal distributions (Fig. 1). HL and HW were positively correlated, suggesting that the husk growth and development were coordinated in the aspects of length and width. The calculation of Broad-sense heritability revealed the high heritability for all the three husk traits (0.91, 0.86, 0.86 for HL, HN and HW, respectively) (Table 1), indicating that the majority of husk phenotypic variations are controlled by genetic factors and suitable for further QTL mapping analysis.

**Table 1:** Variance composition and broad-sense heritability for 191 BC_2_F_8_ families in three environments

Traits	Mo17	RILs		Mean square			Heritability^b^
		Means ± SD	Range	Environment (E)	Genotype (G)	G × E^**a**^	
HL	21.52 ± 1.88	23.49 ± 2.28	17.39–29.80	757.86**	37.24**	3.45**	0.91
NN	9.24 ± 1.92	7.89 ± 0.78	4.80–10.36	5.41**	2.22**	0.31	0.86
HW	7.52 ± 1.00	7.49 ± 0.53	6.22–9.26	40.19**	4.89**	0.7**	0.86

^a^G × E designates the interaction between G and E; ^b^Broad-sense heritability. * *P* ≤ 0.05, ** *P* ≤ 0.01

### Identification of QTLs for three husk traits

**Fig. 2:**
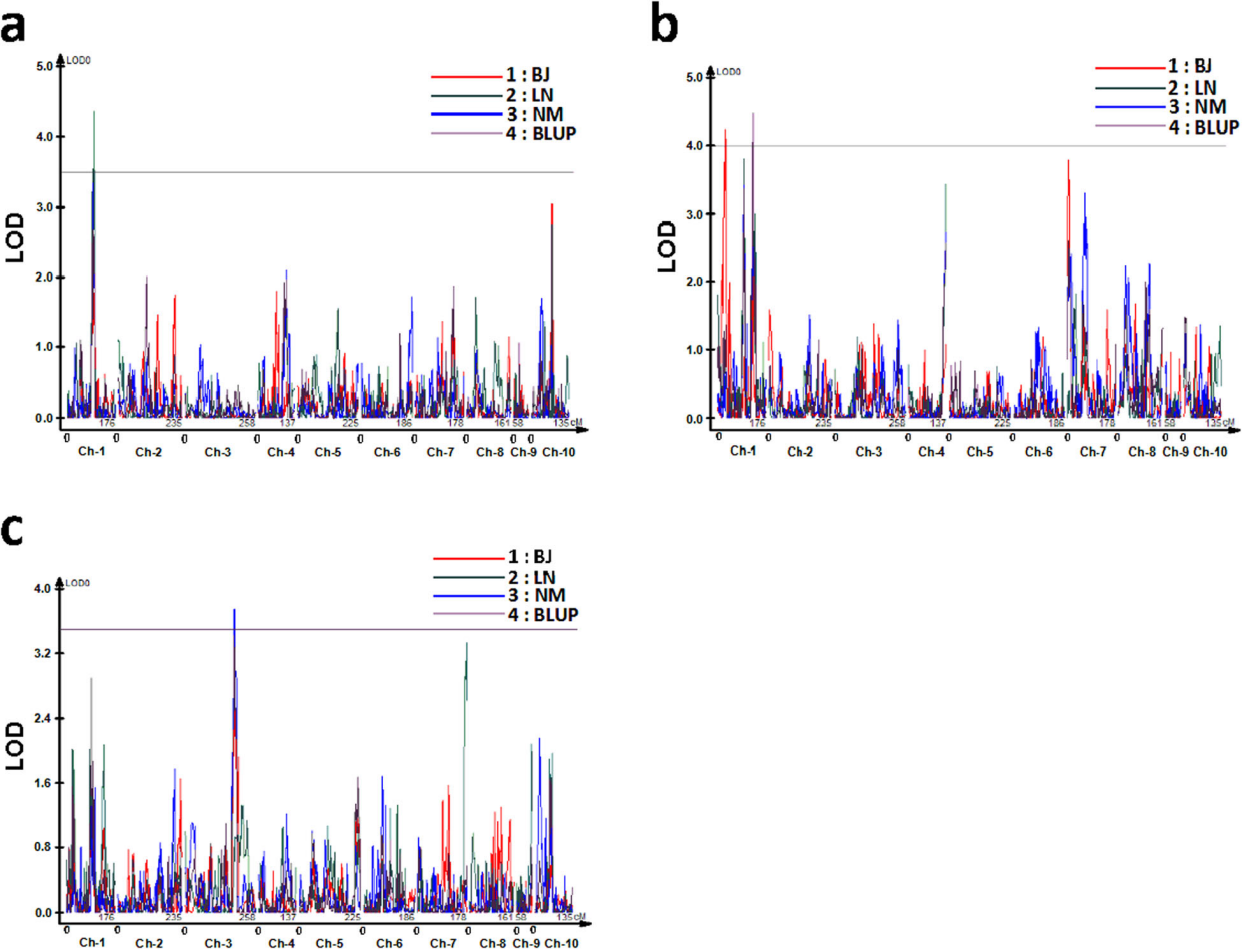
LOD profiles of QTL for three husk traits identified in different environments: **a** HL; **b** HN; **c** HW. BJ, Beijing; NM, Neimeng; LN, Liaoning; BLUP, best linear unbiased prediction

With the ultra-high-density linkage maps, a total of four QTLs were identified after 1000 permutations with an empirical logarithm of the odds (LOD) threshold of 3.5, 4.0 and 3.5 for HL, HN and HW, respectively (Table 2 and Fig. 2). The average of QTL intervals was 6.0 Mb with a range of 5.1–8.9 Mb. For HL, one QTL (qHL-1-1) was detected on chromosome 1 and the phenotypic variation explained by this QTL was 9.6%. For HN, a total of two QTLs (qHN-1-1 and qHN-1-2) were identified on chromosome 1 and the phenotypic variation explained by each individual QTL was 8.9%, respectively. For HW, one QTL (qHW-3-1) was identified on chromosome 3 and explained 8.1% of phenotypic variation. All the four QTLs explained less than 10% of phenotypic variation, indicating that HL, HN and HW are controlled by multiple small-effect QTLs in this BC_2_F_8_ teosinte-maize population.

**Table 2:** Individual QTL for husk traits in the MX BC_2_F_8_ population

Traits	QTL	Environments	Chromosome	Peak (cM)^**a**^	Physical Position (Mb)^**b**^	Genetic interval (cM)	Additive effect^**c**^	LOD value	Phenotypic variation%^**d**^
HL	qHL-1-1	LN	1	97.7	198.5–203.7	93.2–98.2	1.59	4.36	9.6
HN	qHN-1-1	BJ	1	28.1	7.72–16.6	22.8–31.7	−0.25	4.24	8.9
	qHN-1-2	BLUP	1	130.4	275.0–280.1	126.2–131.9	0.29	4.50	8.9
HW	qHW-3-1	NM	3	179.1	204.6–209.8	173.7–183.4	0.30	3.76	8.1

^a^Genetic position in centimorgans (cM) of QTL with the highest LOD; ^b^Physical position of QTL based on the B73 reference sequence (v2); ^c^Additive effect of QTL: a positive value means the allele from the parent Mo17 increases the index of traits, whereas a negative value indicates that the allele from teosinte decrease the index of traits; ^d^ Percentage of the phenotypic variation explained by the additive effect of QTL

### Genetic overlap of QTL in MX and other RIL populations

**Fig. 3:**
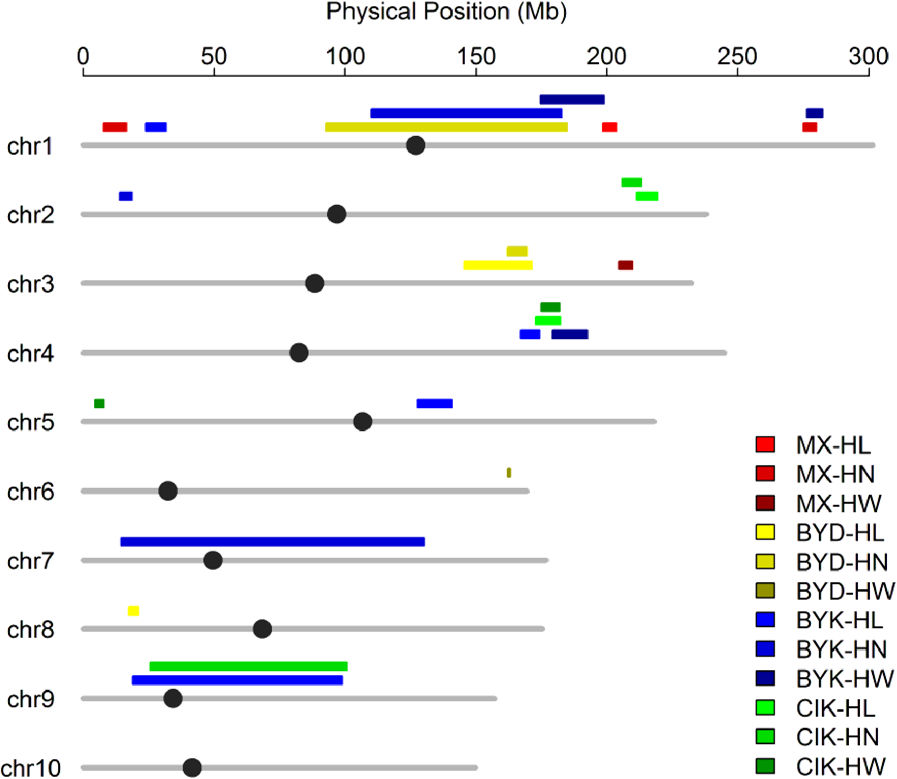
Co-localization of QTLs identified in MX and three other RIL populations

To assess the genetic overlap related to different husk traits, a 1.5-LOD support interval of QTL for HL, HW, and HN in MX population and the other three RIL populations [14] were compared (Fig. 3). This analysis revealed a minimal number of overlap with only qHL-1-1 and qHN-1-2 with a HW QTL in BYK population. These results suggest that genetic loci controlling husk traits in the MX population may largely differ from the other RIL populations.

### Identification of candidate genes and the corresponding tissue-specific expression pattern

**Fig. 4:**
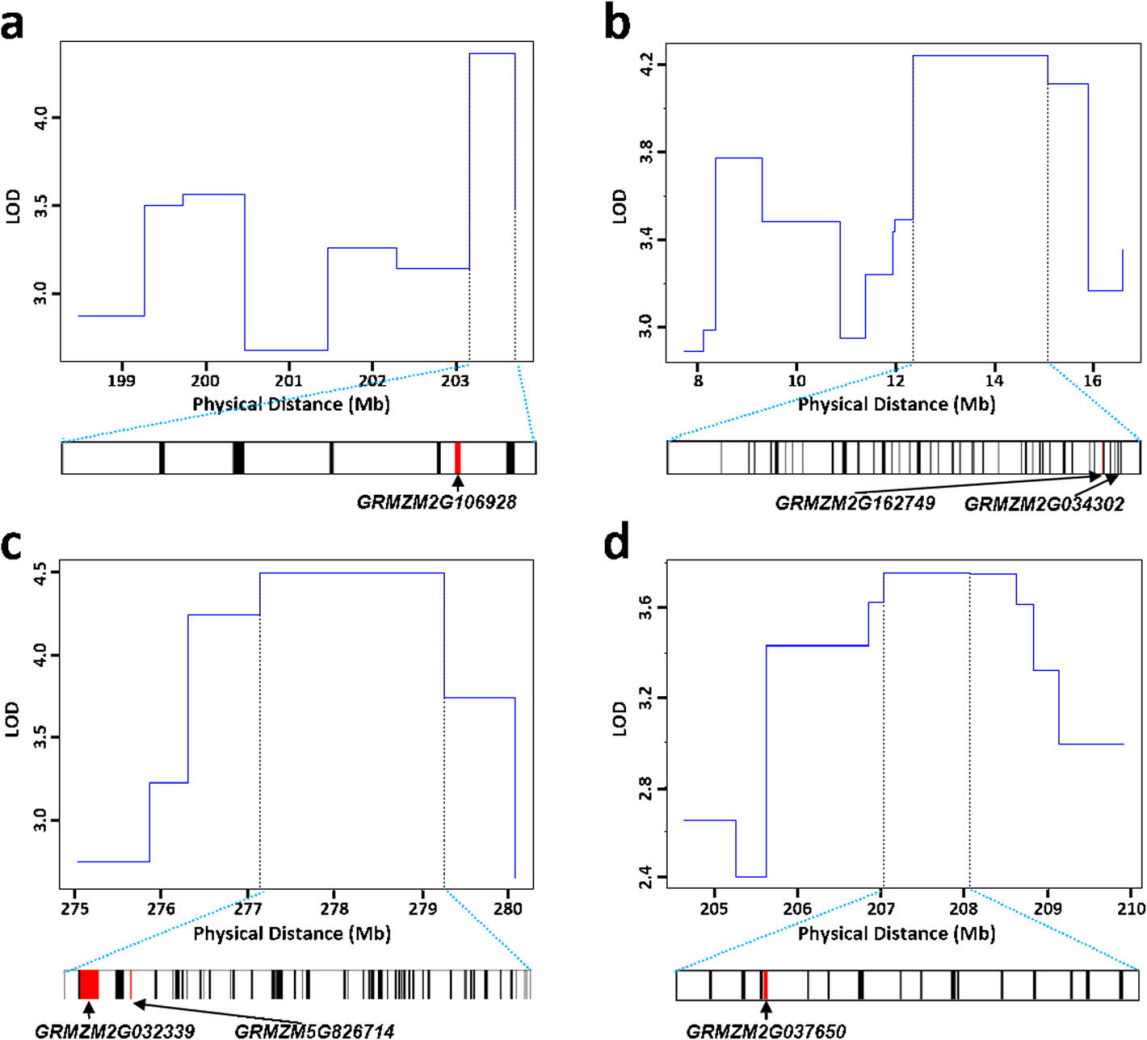
LOD profiles for QTL recombination breakpoints and candidate genes located in the peak points: **a** qHL-1-1; **b** qHN-1-1; **c** qHN-1-2; **d** qHW-3-1. The candidate genes are indicated by red bands and other genes are indicated by gray bands

**Fig. 5:**
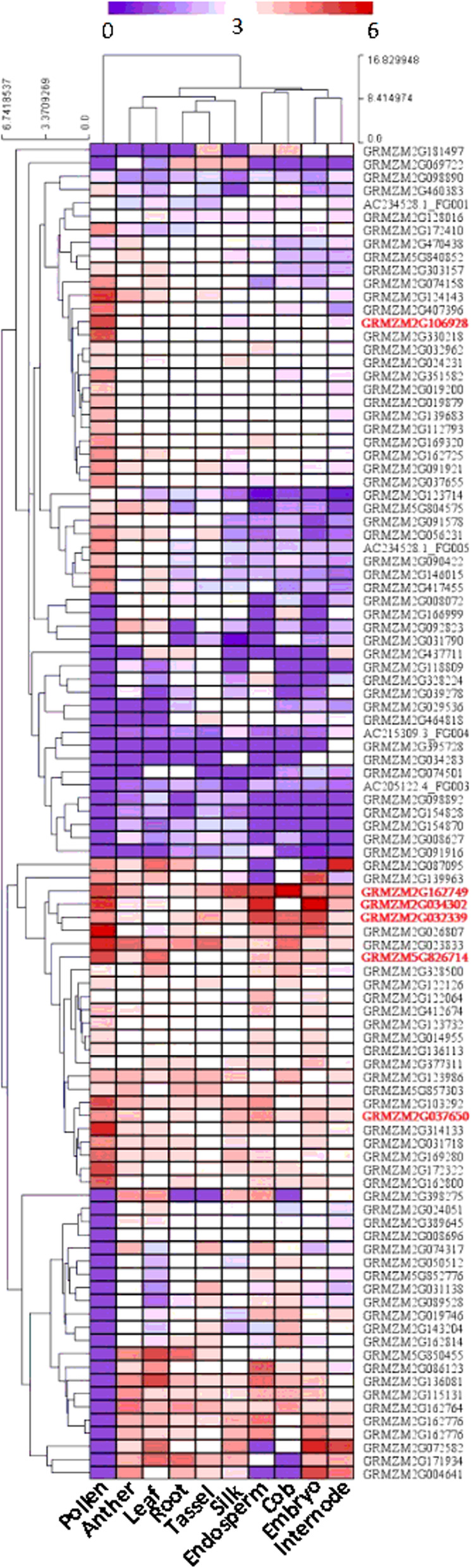
Heat-map showing tissue-specific expression patterns of protein-coding genes within QTL. The log_10_-transformed ratios of normalized RNA-seq counts in husk relative to other tissues as indicated at the bottom of each column. Columns and rows are ordered according to hierarchical cluster analysis at the top and left. The red, white, and blue represent higher, similar or lower expression in husk relative to other tissues, respectively

To explore the candidate genes underlying husk traits, four QTLs were further narrowed by bin map analysis (Fig. 4). The physical distance of peak bins ranged from 0.54 Mb - 2.72 Mb (Table 3). According to the annotation in the MaizeGDB database (www.maizegdb.org), a total of 10, 58, 62 and 16 protein-coding genes were identified within peak bin for qHL-1-1, qHN-1-1, qHN-1-2 and qHW-3-1, respectively. Next, the *in-silico* expression pattern analysis was performed using RNA-seq data collected from husk and other nine different tissues, which are published available in an online comparative RNA-seq expression platform (https://qteller.maizegdb.org) (Fig. 5). Judged from the specific and high expression in husk, a total of six candidate genes with annotated function were identified, including 1, 2, 2 and 1 for HL-1-1, HN-1-1, HN-1-2 and HW-3-1, respectively.

**Table 3:** Candidate genes within the genomic region spanning the single bin

QTL	Chr	Bin	Bin length (bp)	ID	Position^**a**^	Annotation^**b**^
qHL-1-1	1	PZE-101160628	548,905	GRMZM2G106928	203,625,515..203631858	Copper/zinc superoxide dismutase 2
qHN-1-1	1	PZE-101021308	2,719,182	GRMZM2G162749	14,848,652..14854535	Cycling DOF factor 1
				GRMZM2G034302	15,067,584..15075973	Sucrose transporter 2
qHN-1-2	1	SYN9147	2,128,406	GRMZM2G032339	277,216,651..277300425	K-box region and MADS-box transcription factor family protein
				GRMZM5G826714	277,445,149..277451612	COBRA-like extracellular glycosyl-phosphatidyl inositol-anchored protein family
qHW-3-1	3	PZE-103154161	1,040,297	GRMZM2G037650	207,219,415..207228119	Myb domain protein 42

^a^Gene position according to the B73 reference sequence (V2); ^b^Gene annotated according to their homologous gene in *Arabidopsis thaliana* or rice

## Discussion

### Genetic basis of husk traits in the MX population

All the three husk traits in the MX population exhibited a broad range of phenotypic variation with normal distribution. The genetic analysis showed that the heritability of three husk traits is fairly high, indicative of superior genetic effect in this population studied [33]. In addition, except for environment variation, none of significant difference were detected in genetic or interaction between genetic and environment within RILs. Considering this population was conducted by twice back-cross with Mo17, the high consistency of linkage maps among RILs may result in the genetic similarity among MX RILs [34]. Moreover, significantly positive correlation was observed between HL and HW, indicating that the growth and development of husk is coordinated in the dimension of length and width. By comparison, HN was not correlated with HL or HW, suggesting that the molecular pathways regulating the numerous initiations of husk layer may be independent from husk growth.

The husk traits were regulated by one, two and one QTL with small-effects (8.1-9.6%), indicating that each of three husk traits is polygenic and controlled by multiple genes with small effects in the MX population. Interestingly, when compared four QTLs of husk traits in MX with QTLs identified previously in other maize linkage populations [14], we did not detect any overlap of QTLs for the same trait. This result implies that the genetic basis of husk morphology in the MX population is kind of unique compared to other maize linkage population, highlighting the power of the MX population to interpret the genetic variation, which may be never accomplished in the regular modern maize population.

### Candidate genes underlying husk QTLs

To successfully obtain candidate genes, fine-mapping is considered as the general strategy in QTL study. However, it often takes long period for back-cross to get near isogenic lines (NIL) lines. Bin map is an alternative strategy to fine-mapping the yield-associated loci applied in sorghum [35], rice [36], and maize [2, 13, 14, 18]. In this study, four husk related QTLs were narrowed from the original 5.1 Mb - 8.9 Mb interval to 0.54 Mb - 2.72 Mb region according to a bin map. Within four peak bin intervals, there are a total of 102 putative protein-coding genes. By retrieving tissue-specific expression pattern, we could identify six candidate genes with known molecular function and highly expressed in husk. For qHL-1-1, the only candidate gene was GRMZM2G106928, which encodes Copper/zinc superoxide dismutase 2 (Cu/Zn SOD2) involving in the photosynthetic anti-oxidant system [37]. If this gene could be proved by the future functional study, it will provide evident that the photosynthesis may play a role in regulating husk development alike the foliar leaves. For qHN-1-1, two candidate genes GRMZM2G162749 and GRMZM2G034302 were identified, which encode Cycling DOF factor 1 (CDF1) and Sucrose transporter 2 (SUC2), respectively. It has reported in Arabidopsis that Cycling DOF factors are essential for a photoperiodic flowering response [38]. In our previous study, maize flowering time showed significantly positively correlated with HN [13]. In this scenario, it is likely that the maize CDF1 could control HN via mediating the flowering time. It is well known that SUC2 functions in transporting the sucrose into phloem vascular in crops [39]. Therefore, it is likely that sucrose transported by SUC2 plays a role in husk development. For qHN-1-2, two candidate genes GRMZM2G032339 and GRMZM5G826714 were identified, which encode a MADS-box transcription factor and a COBRA-like extracellular glycosyl-phosphatidyl inositol-anchored protein, respectively. It has been reported that the MADS-box transcription factor plays a key role in plant flowering time and node number development [40, 41]. Therefore, this MADS-box transcription factor may also regulate HN through mediating maize flowering time. COBRA-family proteins have been documented as regulators of cellulose biogenesis [42], and act as essential factors in anisotropic expansion via cellulose microfibril orientation of plant morphogenesis [43]. As HN is determined by inner husk organ elongation related to anisotropic expansion, it is conceivable that COBRA-family proteins are involved in the formation of husk. For qHW-3-1, the only candidate gene GRMZM2G037650 encodes a Myb-family transcription factor, which is known to participate in multifaced molecular pathways through regulating down-stream gene expression.

### Importance of QTLs relevant to husk traits in maize genetic and breeding

As the wild ancestor of maize, teosinte exhibits many advantages relative to modern maize, such as significant resistance to biotic or abiotic stresses [23–27]. However, during the maize domestication, hundreds of genes lost. In this context, recovering and utilizing teosinte genes became a promising strategy to further improve modern maize satisfying the requirement of varieties growing in different area. Indeed, a recent study has demonstrated that introgressing the wild UPA2 allele originated from teosinte into modern hybrids could reduce leaf angle, leading to the enhanced yield under high-density condition [44]. The heavy coverage of maize husk offers nursery and healthy environment safeguarding the early stage of ear growth and development. However, it may turn into the major barrier against kernel dehydration after physiological maturation of maize, challenging the mechanical harvest of corn production. Till now, none of genes specially regulating husk development have been identified, raising a critical issue that we do not have any objective to fulfil gene editing. Therefore, the husk-relevant QTLs offers prospective routes to modify husk morphology through molecular marker-assisted selection in maize breeding program.

## Conclusion

In this work, we describe the interpretation of the genetic basis and QTL mapping of three husk traits in a teosinte-maize population. A total of four QTLs underlying husk length and width as well as the number of husk layer were identified. Importantly, all four QTLs were not overlapped with other husk-relevant QTLs identified in the previous population. Therefore, the newly-identified QTLs in this study will greatly enlarge genomic targets to explore candidate genes regulating husk growth and development, and benefit the breeding program based on molecular marker-assisted selection to pursue new varieties with proper husk morphology.

## Methods

### Plant materials and phenotyping

The maize-teosinte (Mo17/X26–4, MX) RIL population including 191 families was derived from crossing between Mo17 and one teosinte line (Teo X26–4, accession number PI 566686). The F_1_ individual was backcrossed with Mo17 twice and then self-pollinated for eight generations lead to the construction of the maize-teosinte introgression BC_2_F_8_ population. It is noted that both of parent lines, Mo17 and teosinte were originally obtained from the maize stock center (http://maizecoop.cropsci.uiuc.edu/), and the detailed information about the development of the MX population has been described in two previous studies [34, 45]. The MX population was planted in a randomized complete block design at three different regions including Beijing (BJ, 40°08′N, 116°10′E), Neimeng (NM, 4031′N, 107°05′E), and Liaoning (LN, 40°’82’N, 123°56′E) in 2015 and 2016. Each line was grown in a single-row plot with a row length of 250 cm and 60 cm between rows under natural field conditions. The details about husk trait measurement were described previously [13]. We declare that all the collections of plant and seed specimens related to this study were performed in accordance with the relevant guidelines and regulations by Ministry of Agriculture (MOA) of the People’s Republic of China.

### Analysis of phenotypic data

The phenotypic variation of husk traits was analyzed using R software 4.0.1 with the “aov” function (ANOVA). The model for the ANOVA was y = + i + j +, where i is the effect of ith genotype, j is the effect of the jth environment with error. The broad-sense heritability of husk traits was calculated as: h2 = G2/(G2 + GE2/n + 2/n) [46], where G2 is genetic variance, GE2 is the interaction of genotype with environment, 2 is the resident error and n is the number of the environments. The BLUP value was calculated using a linear mixed model. Both genotype and environment were treated as random effects in the R function “lme4”.

### Genotyping and constructing the bin map

The genotype of the MX population was obtained by utilizing the Illumina MaizeSNP50 array (Illumina Inc., San Diego, CA, USA) [47], containing 56,110 SNPs. Quality control was performed by removing monomorphic markers (MAF < 5%) with a missing rate higher than 10% by PLINK software [48]. Finally, 12,390 high-quality SNPs were selected to build the genetic linkage map with CarthaGene software [49] using Perl scripts in a Linux system (www.maizego.org/Resources.html). The details about the construction of genetic linkage maps has been described previously [34]. The co-segregating markers were merged into a bin. With the logarithm of the odds (LOD) of each bin marker, a bin map could be drawn following the physical position of bin marker.

### QTL mapping

The QTLs were analyzed by composite interval mapping method implemented in Windows QTL Cartographer 2.5 [50]. Genome was scanned at every 1.0 cM interval between markers using a 10 cM window size. A forward and backward stepwise regression with five controlling markers was conducted to control background from flanking markers. The LOD threshold was determined by the 1000 permutations at a significance (*P* < 0.05) and used to identify the significant QTL [51]. With the 1.5-LOD support interval method, the confidence interval for each QTL position was estimated [52].

### Gene annotation

QTLs were delimited to a single peak bin interval based on bin map. The protein-coding genes within intervals were listed according to MaizeGDB database (V2). Each of the corresponding gene were annotated by performing BLASTP searches at the NCBI (blast.ncbi.nlm.nih.gov/Blast.cgi).

### Tissue-specific expression pattern of candidate genes

RNA-seq dataset from husk were downloaded from NCBI’s Sequence Read Archive (SRA) database. The quality of RNA-seq reads were controlled by FastQC software. Sequence reads were aligned to B73 RefGen_v2 by the TopHat (v2.1.0) pipeline with a built-in Bowtie (v0.12.9) program. Unique-mapped reads were retained for counting FPKM. All the RNA-seq datasets from other nine tissues were obtained by qTeller RNA-seq expression platform (https://qteller.maizegdb.org). Then the FPKM was calculated to TPM by the model:

1 $$ TPMi=\left(\frac{FPKM}{\sum \limits_j FPKMj}\right)\times {10}^6 $$

Values used in heat-map plot were the log_10_-transformed ratios of normalized TPM counts in husk relative to other tissues.

## Data Availability

Genotyping and sequencing data from this study can be found in the GenBank database under accession number MN026742–MN026862.
